# Non-invasive neuromodulation for drug-resistant epilepsy: a contemporary structured review of mechanisms, clinical evidence and future directions

**DOI:** 10.3389/fneur.2026.1950268

**Published:** 2026-09-18

**Authors:** Daniel San-Juan, Ximena Blasco, Emmanuel Polito-Salazar, Alejandro Velasco-Hernandez, Hector Francisco Galindo-Bustamante, Giovanni Sousa-Oliva, Norberto Agustín Toledo-De la Rosa, Antonio Valentín, Ioannis Stavropoulos

**Affiliations:** 1 Epilepsy Clinic, National Institute of Neurology and Neurosurgery, Mexico City, Mexico; 2 Epilepsy Clinic, Hospital Angels of Pedregal, Mexico City, Mexico; 3 Faculty of Medicine, Autonomous University of Puebla, Puebla, Mexico; 4 Faculty of Medicine, National Autonomous University of Mexico, Mexico City, Mexico; 5 National School of Medicine and Homeopathy, National Polytechnic Institute, Mexico City, Mexico; 6 Department of Basic and Clinical Neuroscience, IoPPN, King’s College London, United Kingdom; 7 Department of Clinical Neurophysiology, King’s College Hospital, London, United Kingdom

**Keywords:** drug-resistant epilepsy, non-invasive neuromodulation, transcranial direct current stimulation, transcranial magnetic stimulation, transcutaneous vagus nerve stimulation

## Abstract

Drug-resistant epilepsy (DRE) affects nearly one-third of people with epilepsy and remains a major cause of disability despite continued advances in antiseizure medications and surgical therapies. For patients who are not candidates for resective surgery or invasive neuromodulation, non-invasive neuromodulation (NIN) has emerged as a promising strategy to modulate epileptic networks while avoiding the risks associated with implanted devices. This structured narrative review examines current evidence across the principal NIN modalities, including repetitive transcranial magnetic stimulation (rTMS), transcranial electrical stimulation (transcranial direct current (tDCS), alternating current, and temporal interference stimulation), transcutaneous vagus nerve stimulation, trigeminal nerve stimulation, and transcranial focused ultrasound. We integrate current knowledge on mechanisms of action, clinical efficacy, safety, and practical considerations while highlighting recent advances that may shape future clinical implementation. Available evidence indicates that low-frequency rTMS and cathodal tDCS provide the most consistent reductions in seizure frequency and interictal epileptiform discharges, particularly in focal DRE. Transcutaneous vagus nerve stimulation has shown encouraging results with an excellent safety profile, whereas focused ultrasound, trigeminal nerve stimulation, and temporal interference stimulation remain investigational and require further clinical validation. Across modalities, interpretation of current evidence is limited by heterogeneous stimulation protocols, small study populations, and variable outcome measures. Emerging approaches, including individualized targeting, connectome-guided stimulation, computational modeling, neurophysiological biomarkers, and adaptive closed-loop neuromodulation, are expected to improve treatment precision and patient selection. Although high-quality multicenter randomized trials remain necessary, current evidence supports NIN as an increasingly relevant component of personalized epilepsy care and a promising avenue for expanding therapeutic options in DRE.

## 1 Introduction

Globally, epilepsy affects an estimated 51.7 million people, corresponding to approximately 0.7% of the population with active disease, while nearly 3.3 million individuals develop new-onset epilepsy each year (1). Despite major advances in anti-seizure medications (ASMs), approximately one-third of people with epilepsy (PWE) develop drug-resistant epilepsy (DRE), one of the leading causes of epilepsy-related morbidity (2). The International League Against Epilepsy (ILAE) defines DRE as the failure of adequate trials of two appropriately selected, tolerated, and correctly administered ASMs, whether used as monotherapy or in combination, to achieve sustained seizure freedom (3).

For appropriately selected patients, surgical resection remains the most effective treatment, achieving seizure freedom in 58–73% of individuals with lesional temporal lobe epilepsy (TLE), compared with only 0–8% receiving medical therapy alone (4, 145). However, outcomes are considerably less favorable in non-lesional or extratemporal epilepsy. In addition, epilepsy surgery has important limitations, including high upfront costs, the risk of permanent neurological deficits, postoperative infections, and uncertain long-term cognitive and psychiatric outcomes (5, 6). For patients who are not candidates for resective surgery or who continue to experience seizures after surgery, FDA-approved invasive neuromodulation therapies, including vagus nerve stimulation (VNS), anterior thalamic deep brain stimulation (ANT-DBS), and responsive neurostimulation (RNS), provide established therapeutic alternatives. Long-term follow-up studies demonstrate progressive improvements in efficacy, with responder rates reaching 73–75% after 7–9 years while maintaining favorable safety profiles and reducing the risk of sudden unexpected death in epilepsy to approximately 2.0 deaths per 1,000 person-years (7, 8). Nevertheless, implantation-related complications, including pain and device-site infection, occur in 7.3–12.7% of patients, whereas intracranial procedures carry an additional risk of non-seizure-related hemorrhage (9). Stimulation-induced adverse effects, such as dysphonia following VNS or mood and memory disturbances associated with intracranial stimulation, are also relatively common but generally remain stable over time (7, 10).

Against this background, non-invasive neuromodulation (NIN) has emerged as a promising therapeutic strategy with the potential to improve seizure control while avoiding the risks associated with surgical implantation. Recent systematic reviews and meta-analyses provide important quantitative estimates for individual NIN modalities; however, their scope is generally modality-specific, and differences in stimulation protocols, patient populations, treatment schedules, and outcome definitions limit direct clinical interpretation across approaches (11, 12).

Our structured narrative review complements these quantitative syntheses by integrating evidence across rTMS; transcranial electrical stimulation (tES), including transcranial direct current stimulation (tDCS), transcranial alternating current stimulation (tACS), transcranial random noise stimulation (tRNS), and transcranial temporal interference stimulation (tTIS); transcutaneous vagus nerve stimulation (tVNS), comprising transcutaneous auricular (taVNS) and transcutaneous cervical (tcVNS) approaches; transcranial focused ultrasound (tFUS); and trigeminal nerve stimulation (TNS), with a particular emphasis on mechanistic principles, stimulation parameters, clinical efficacy, safety, and neurophysiological and network-level biomarkers. We incorporate recent primary studies published after the latest evidence syntheses and critically examine the relationship between electrophysiological effects and clinical outcomes, the heterogeneity of stimulation protocols, and the strength and limitations of the evidence supporting each modality. We also examine emerging precision neuromodulation strategies, including individualized targeting, computational electric-field modeling, SEEG-guided stimulation, connectome-informed approaches, multichannel electric-field shaping, and adaptive closed-loop paradigms, and consider how these approaches may address current limitations and improve patient selection and treatment delivery. Thus, rather than replacing modality-specific systematic reviews and meta-analyses, this review provides a complementary clinical and translational synthesis that integrates their findings across the broader landscape of NIN for DRE.

## 2 Materials and methods

### 2.1 Study design

This structured narrative review provides a comprehensive, clinically oriented synthesis of the current evidence on NIN for DRE.

This study design was selected over a systematic review with meta-analysis because the available literature is characterized by substantial clinical and methodological heterogeneity. Studies differ considerably in stimulation targets, study design, protocols, mechanisms of action, patient populations, study designs, follow-up duration, and outcome measures, including seizure response definitions. Consequently, quantitative pooling across modalities would be methodologically inappropriate and could generate misleading estimates.

Accordingly, this review adopts a structured narrative framework to critically synthesize the available evidence, identify consistent efficacy patterns, discuss shared and modality-specific mechanisms, evaluate safety profiles, and highlight current knowledge gaps and future research priorities.

Methodological transparency and reporting quality were guided by the Scale for the Assessment of Narrative Review Articles (SANRA) (13), a validated framework that strengthens literature justification, critical appraisal, transparency, and balanced interpretation in narrative reviews.

### 2.2 Search strategy

A structured literature search was conducted in PubMed/MEDLINE, Embase, Scopus, and Web of Science from database inception through April 15, 2026. The search combined Medical Subject Headings (MeSH) and free-text terms related to epilepsy, DRE, and NIN techniques, including rTMS, tES (tDCS, tACS, and tRNS), tVNS, TNS, and tFUS. Whenever supported by the database, filters were applied to exclude animal-only studies and case reports. The complete search strategies for each database are provided in the Supplementary material.

Reference lists of all eligible systematic reviews, meta-analyses, RCTs, and key review articles were manually screened to identify additional relevant publications that were not captured during the electronic search. Citation tracking was also performed for landmark studies and recent systematic reviews to ensure comprehensive coverage of the available literature.

Although a comprehensive search strategy was employed to maximize identification of relevant literature, the present work was designed as a narrative review rather than a systematic review. Consequently, the retrieved evidence was synthesized to provide a clinically oriented, mechanistic, and methodologically informed overview of NIN for DRE, rather than through formal systematic review methodology.

### 2.3 Eligibility criteria study selection

Eligible studies investigated one or more NIN techniques for epilepsy, enrolled pediatric or adult patients with DRE whenever defined according to contemporary ILAE criteria, and reported original clinical or neurophysiological outcomes. To ensure clinical relevance, studies were required to report at least one meaningful outcome, including seizure frequency, responder rate (≥50% seizure reduction), seizure freedom, quality of life, adverse events, interictal epileptiform discharges, EEG biomarkers, cortical excitability, functional connectivity, or other network-level neurophysiological measures. Only full-text, peer-reviewed articles published in English were considered.

Systematic reviews and meta-analyses were prioritized as the highest level of evidence for each neuromodulation modality. Primary clinical studies published after the most recent evidence syntheses were subsequently incorporated when they contributed novel efficacy, safety, or mechanistic data.

Studies were excluded if they exclusively evaluated invasive neuromodulation, enrolled healthy volunteers or non-epileptic populations, consisted of animal or *in vitro* research, or lacked sufficient methodological detail, including conference abstracts, editorials, letters, expert opinions, narrative commentaries, study protocols, and case reports. Duplicate publications, secondary analyses without additional original data, and studies focused solely on device development without clinical or neurophysiological outcomes were also excluded.

### 2.4 Evidence hierarchy and qualitative appraisal

Systematic reviews and meta-analyses were considered the principal sources of evidence and were prioritized whenever available. When multiple reviews addressed the same intervention, preference was given to the most recent and comprehensive publications, considering their relevance to the review objectives, scope of included evidence, and clarity of reporting.

RCTs constituted the primary source of original evidence for evaluating clinical efficacy and safety. Prospective and retrospective cohort studies, together with comparative observational studies, were included to complement randomized evidence, particularly for longer follow-up periods, underrepresented populations, or recently developed techniques not yet incorporated into quantitative reviews. Case series were considered only when higher-level evidence was unavailable or when describing emerging technologies and novel stimulation paradigms.

Rather than applying formal risk-of-bias instruments, studies underwent structured qualitative appraisal based on study design, sample size, patient selection, control group adequacy, randomization, blinding, follow-up duration, completeness of outcome reporting, reproducibility, and external validity. Greater interpretive weight was assigned to higher-quality evidence, whereas findings from uncontrolled observational studies and case series were interpreted within the context of their inherent methodological limitations.

### 2.5 Data extraction and narrative evidence synthesis

Data were extracted using a standardized framework capturing clinical, methodological, and mechanistic characteristics of non-invasive neuromodulation (NIN) studies. Clinical outcomes included seizure frequency reduction, responder rates, seizure freedom, durability of treatment response, and safety, while secondary outcomes encompassed neurophysiological biomarkers, cortical excitability, functional connectivity, cognition, and quality of life. Owing to substantial heterogeneity in study designs, patient populations, stimulation protocols, and outcome reporting, findings were synthesized qualitatively and organized by neuromodulation modality and thematic domains. Quantitative efficacy metrics were incorporated whenever consistently reported to enhance clinical relevance. Direct cross-modality comparisons were deliberately avoided because of methodological and clinical heterogeneity, providing a balanced and evidence-based overview of the current literature.

## 3 Neuromodulation techniques

### 3.1 Transcranial magnetic stimulation

#### 3.1.1 Introduction and historical background

The origins of TMS can be traced to nineteenth-century discoveries in electromagnetism, culminating in a major breakthrough in 1985 when Anthony Barker and colleagues developed the first device capable of noninvasively stimulating the human motor cortex through the intact scalp. Their landmark study demonstrated that magnetic stimulation could reliably evoke contralateral muscle contractions, establishing TMS as a practical tool for investigating cortical physiology and laying the foundation for modern brain mapping and therapeutic neuromodulation (14, 15).

Throughout the 1990s and early 2000s, clinical studies demonstrated the therapeutic potential of rTMS, leading to FDA approval for treatment-resistant major depressive disorder in 2008, its first and most established clinical indication. Subsequent approvals expanded its use to obsessive-compulsive disorder, migraine with aura, nicotine dependence, and anxiety symptoms associated with depression. Parallel advances in coil design and stimulation paradigms, particularly theta-burst stimulation, have further improved treatment efficiency and broadened clinical applications. Despite these advances, most neurological and psychiatric indications, including epilepsy, remain investigational (16–19).

#### 3.1.2 Principles and mechanism of action

TMS is based on the principle of electromagnetic induction, whereby rapidly changing magnetic fields generated by an external stimulation coil induce electric currents within cortical tissue, leading to neuronal depolarization and modulation of cortical excitability (20).

Although the biophysical principles underlying TMS are well established, the downstream neurophysiological mechanisms remain only partially understood (21, 22). Beyond direct neuronal depolarization, stimulation induces complex cellular and synaptic responses that are influenced by cortical state, functional connectivity, and ongoing network activity (23, 24). Current evidence suggests that both mechanisms contribute, with stimulation effects influenced by functional connectivity and brain-state dependency (21, 25, 26). This incomplete mechanistic understanding may partly explain variability in clinical responses.

Magnetic pulses can trigger synaptic changes resembling long-term potentiation (LTP) and long-term depression (LTD), resulting in sustained alterations in cortical excitability and functional connectivity (21, 27). TMS effects are not restricted to the targeted cortex but also extend to anatomically and functionally connect regions (26, 28). Consequently, contemporary neuromodulation research increasingly adopts a network-based perspective, recognizing that stimulation frequency, pulse pattern, coil geometry, electric field distribution, and targeting accuracy collectively determine therapeutic responses (29, 30). Advances in computational modeling and coil engineering have further improved electric field precision, facilitating individualized stimulation strategies (21, 31, 32). Experimental studies also suggest broader biological effects involving neurotrophic signaling, glial modulation, neurogenesis, and neuroprotection (33).

In epilepsy, the therapeutic rationale is based primarily on the inhibitory effects of low-frequency rTMS (≤1 Hz) applied over the epileptogenic cortex (Figure 1). By reducing cortical hyperexcitability, low-frequency stimulation may suppress synchronized epileptiform discharges, increase seizure threshold, and ultimately decrease seizure susceptibility (34). Because epileptic activity involves distributed networks, stimulation may also modify connectivity in distant regions involved in seizure propagation (25).

**Figure 1:**
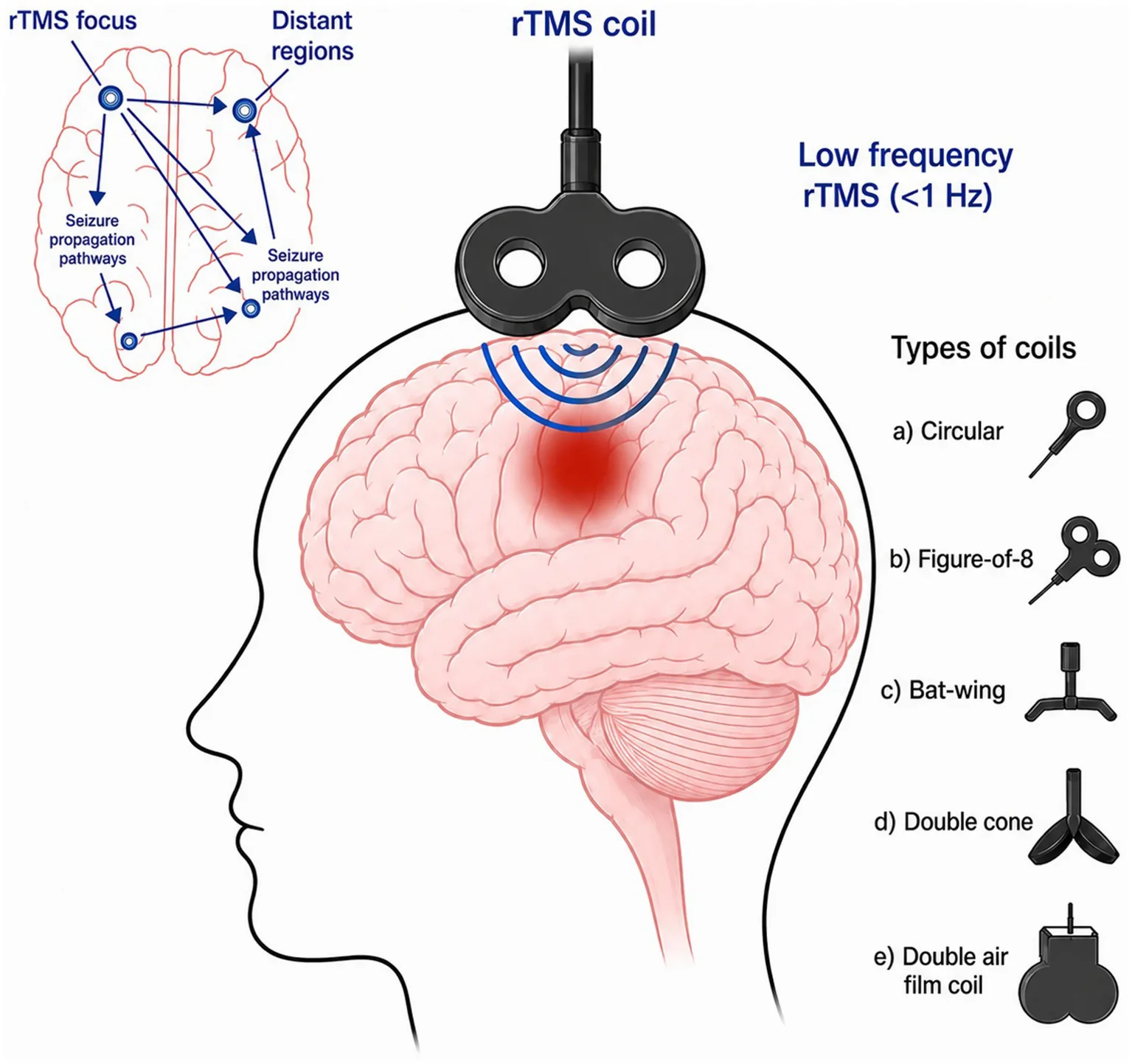
Therapeutic mechanisms of low-frequency rTMS, ≤1 Hz in epilepsy. The coil is placed directly over the scalp and generates brief magnetic pulses. Different coil designs, such as figure-of-eight coils, circular coils, etc. (examples of different coil types are shown on the right) determine the focality and depth of stimulation. Figure-of-eight coils are most commonly used because they provide more precise cortical targeting. Inhibitory stimulation applied over the epileptogenic cortex reduces cortical hyperexcitability, decreasing synchronized epileptiform activity and seizure susceptibility. Additionally, rTMS may modulate connectivity in distant seizure-related networks, influencing broader seizure propagation pathways (top left).

#### 3.1.3 Stimulation protocols and technical parameters

Commonly rTMS used frequencies range from 0.3 to 1 Hz, with stimulation intensity typically set at 90–100% of the resting motor threshold (35–37). The number of pulses per session varies widely across studies (500 to 1,500 pulses) (38). Treatment courses typically involve daily sessions over 5–10 days, although longer or accelerated protocols have also been reported (36, 37, 39).

Accurate localization of the epileptogenic cortex is considered one of the principal determinants of treatment efficacy. Most studies target cortical regions identified by EEG or neuroimaging, whereas others stimulate broader cortical areas, such as the vertex, to modulate large-scale epileptic networks. More recently, functional connectivity and network-based targeting strategies have emerged, reflecting the transition from focal stimulation toward connectome-guided neuromodulation (40). The figure-of-eight coil is commonly used due to its focal stimulation properties, although round coils have also been employed without clear efficacy differences (38, 41).

Clinical benefits generally persist for several weeks and may extend to approximately two months in selected patients. However, treatment durability remains variable, and evidence is currently insufficient to establish standardized maintenance protocols for DRE (38, 39, 41).

#### 3.1.4 Clinical evidence in DRE

Clinical evidence supporting rTMS in DRE remains heterogeneous (Table 1). An updated Cochrane review included eight randomized controlled trials involving 241 participants. Two trials reported significant reductions in seizure frequency from baseline, while the remaining studies did not demonstrate significant differences compared with controls. Of four studies evaluating epileptiform discharges, three reported significant reductions following active rTMS; however, substantial heterogeneity prevented quantitative pooling, and the certainty of evidence for seizure reduction remained low to very low (42).

**Table 1:** Clinical application of rTMS by population.

Population	Clinical application
Pediatrics	rTMS emerging evidence suggesting greater responsiveness in younger patients. In a recent sham controlled randomized trial involving 49 children with focal DRE, 76% of patients in the active rTMS group achieved a statistically significant responder rate in seizure frequency, compared to 12.5% in the sham group. In addition, active rTMS was associated with significant improvements in cognitive and behavioral outcomes, as well as a reduction in IEDs on sleep EEG, at 8 weeks post therapy (136). These findings support the role of targeted low frequency rTMS as a promising adjunctive therapy in children with focal DRE, with both clinical and neurophysiological benefits. In a systematic review and individual participant data meta-analysis of real-world evidence, rTMS demonstrated a particularly favorable response in younger populations. Among patients aged <21 years, the pooled analysis showed a median seizure reduction of 69% (49–84%) (137). Despite these encouraging findings, the evidence base for rTMS in pediatric DRE remains limited. Most available data derive from small cohorts or mixed age populations, with relatively few studies specifically designed for children.
Adults	Across randomized and controlled studies included in a recent systematic review, the effect of rTMS on seizure frequency showed considerable variability, with responder rates ranging from 0% to approximately 80% in active treatment groups. The most robust effect was observed in selected cohorts, where up to 83% of patients achieved ≥50% seizure reduction, including a subset who became seizure-free. However, in other trials, responder rates were more modest, generally around 20–30%, and in some studies no significant difference was observed compared to sham stimulation. Overall, these findings suggest a heterogeneous but potentially meaningful clinical effect, highly dependent on stimulation parameters, patient selection, and study design. Also, rTMS was associated with a reduction in IEDs across studies, with reported decreases ranging from approximately 16 to 65%. Some studies demonstrated early and pronounced reductions within the first 24 h, while others showed more moderate or sustained effects over several weeks (42). Outcomes are variable and evidence insufficient for routine use.
Focal epilepsy	rTMS in focal epilepsy have modest and heterogeneous efficacy, with about 30% of patients achieving ≥50% seizure reduction. Outcomes appear more favorable in focal, well-localized epilepsy, particularly when targeted low-frequency stimulation is guided by EEG, MRI, or neuronavigation. Factors such as temporal lobe focus, use of figure-8 coils, and precise targeting are associated with better responses, while evidence from randomized trials suggests reductions in seizures and IEDs in selected focal lesions (137, 138).
Generalized epilepsy	Current evidence does not support rTMS as an effective therapy for primary generalized epilepsies, with limited and inconsistent data largely derived from studies focused on focal epilepsy. While rTMS appears generally safe and well tolerated (138).

Among individual trials, Fregni et al. (43) reported a 72% reduction in seizure frequency, with IED reductions of 31% immediately after treatment and 16% at 4 weeks. Sun et al. reported a 78.9% reduction in seizure frequency and a 65.8% reduction in IEDs after high-intensity stimulation (39), while Joo et al. (44) observed a 54.9% reduction in IEDs. More recently, an updated meta-analysis reported pooled seizure-frequency reductions of 49 and 19% at four and 8 weeks, respectively (45). In a 2025 sham-controlled crossover trial, low-frequency rTMS produced a numerical reduction in IEDs, although the difference was not statistically significant (46).

Overall, rTMS appears to have a favorable safety profile and may produce measurable electrophysiological effects, but its sustained clinical antiseizure efficacy remains uncertain. Interpretation is limited by small samples, heterogeneous stimulation protocols and targeting strategies, and limited long-term follow-up.

### 3.2 Transcranial electrical stimulation

#### 3.2.1 Introduction and historical background

Transcranial electrical stimulation (tES) comprises a family of non-invasive neuromodulation techniques that deliver low-intensity electrical currents through scalp electrodes to modulate cortical excitability, synaptic plasticity, and large-scale brain network activity (47).

The therapeutic use of electrical stimulation dates back more than two millennia, from the empirical application of torpedo fish described by Scribonius Largus to the pioneering electrophysiological experiments of Giovanni Aldini in the nineteenth century (48–50). Modern interest in tES was renewed following the landmark demonstration by Nitsche and Paulus that tDCS induces polarity-dependent modulation of cortical excitability, providing the first reproducible evidence that weak direct currents can produce sustained neurophysiological effects beyond the stimulation period (51). This discovery established the foundation for the clinical development of tES and stimulated growing interest in its application to DRE, where modulation of cortical excitability and epileptic networks offers a potential non-pharmacological therapeutic strategy (52, 53).

#### 3.2.2 Transcranial direct current stimulation: mechanism of action

In epilepsy, cathodal tDCS (c-tDCS) is predominantly used to reduce cortical hyperexcitability by inducing membrane hyperpolarization, thereby increasing the threshold for neuronal firing and suppressing IEDs (Figure 2) (54). Experimental studies further indicate that c-tDCS enhances GABAergic inhibition within the epileptogenic cortex while promoting activity-dependent plasticity through NMDA receptor-dependent mechanisms resembling LTP and LTD (53, 55–57). Beyond local effects, stimulation reshapes functional connectivity and may disrupt the pathological synchronization underlying seizure generation and propagation (58–61). Acute and long-term effects of c-tDCS are summarized in Table 2.

**Figure 2:**
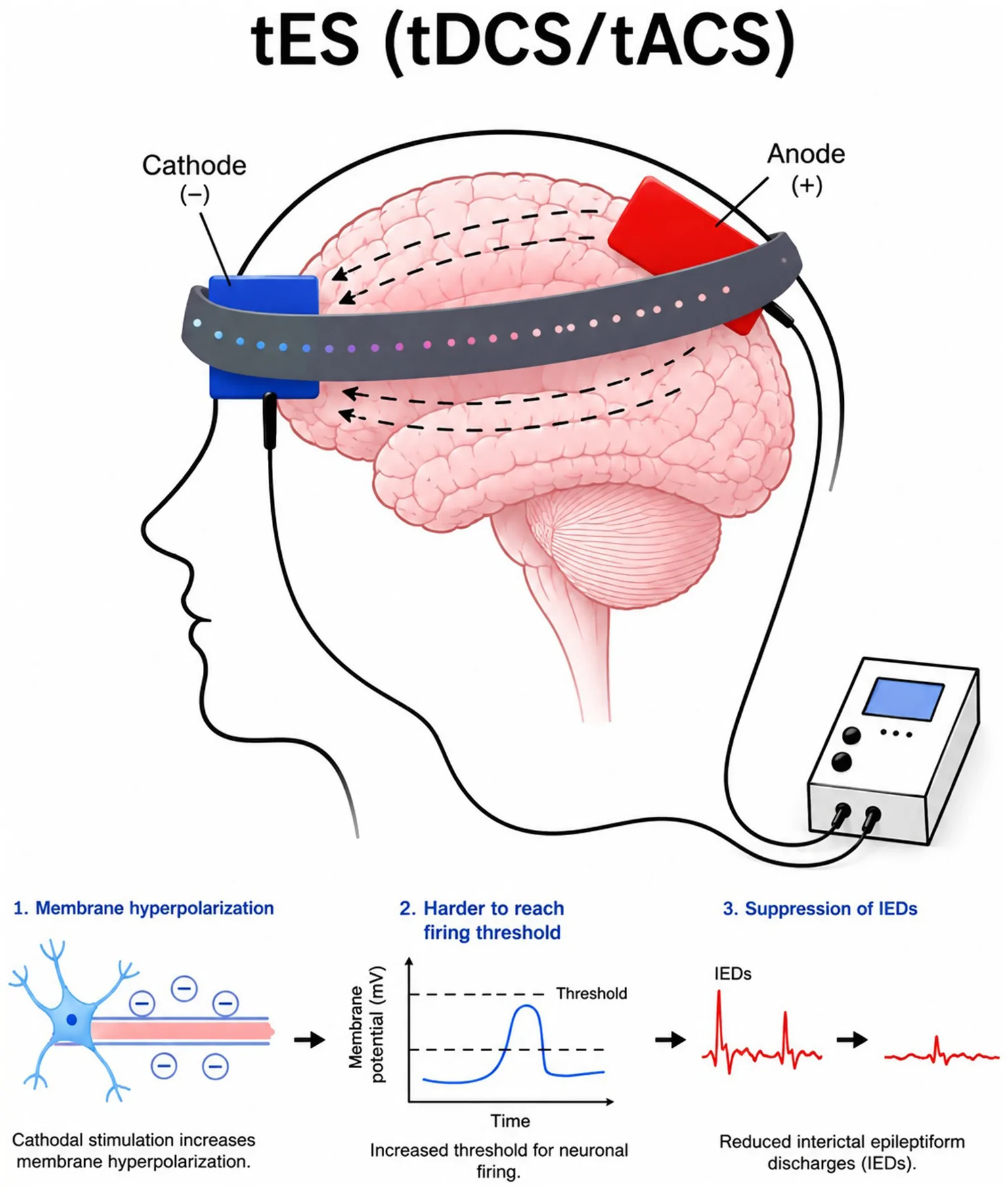
This figure demonstrates an example of transcranial electrical stimulation techniques, including tDCS and tACS, in epilepsy. Electrodes are placed on the scalp over specific cortical regions. One electrode functions as the anode (+) and the other as the cathode (−). A headband secures the electrodes in place to ensure stable contact with the scalp and consistent current delivery during stimulation sessions. Electrode placement determines the direction and distribution of current flow through the brain. Cathodal stimulation reduces cortical excitability through membrane hyperpolarization, increases the neuronal firing threshold, and suppresses IEDs.

**Table 2:** Acute and long-term effects of c-tDCS.

Domain of effect	Acute effect	Long-term effect	Reference
Cortical excitability modulation	Immediate polarity-dependent changes in neuronal membrane potential; anodal stimulation increases excitability and cathodal stimulation decreases neuronal firing probability.	Persistent changes in cortical excitability due to activity-dependent synaptic plasticity after repeated stimulation sessions.	(51)
Synaptic plasticity	Subthreshold membrane polarization alters synaptic efficacy and neuronal responsiveness.	Long-lasting plasticity mechanisms involving glutamatergic synapses and NMDA-dependent pathways may maintain neuromodulatory effects.	(122)
Network connectivity and synchronization	Rapid modulation of functional connectivity and synchronization within cortical networks detectable by EEG.	Longer-term reorganization of epileptic networks and functional connectivity patterns may occur after repeated stimulation.	(53)
Brain oscillatory activity	tACS and other tES modalities can entrain or disrupt ongoing neuronal oscillations and influence rhythmic brain activity.	Repeated stimulation may induce durable changes in oscillatory patterns and network synchronization.	(139)
Neurotransmitter modulation	Acute shifts in excitatory–inhibitory balance through modulation of glutamate and GABA signaling.	Persistent modulation of neurotransmitter systems and inhibitory tone may contribute to therapeutic effects in epilepsy.	(140)
Cognitive and behavioral effects	Temporary changes in attention, perception, or cognitive processing depending on stimulation site.	Evidence for durable cognitive improvement is inconsistent; most epilepsy trials show minimal long-term cognitive changes.	(118)
Mood and neuropsychiatric symptoms	Acute modulation of mood circuits in the prefrontal cortex.	Sustained improvements in mood or anxiety symptoms reported in some clinical neuromodulation studies.	(141)
Safety and tolerability	Mild transient adverse effects including tingling, itching, erythema, or mild headache during stimulation.	Long-term adverse effects are rare when stimulation parameters remain within recommended safety limits.	(62)

##### 3.2.2.1 Transcranial direct current stimulation: stimulation parameters

Standard c-tDCS protocols typically deliver 1–2 mA for 20–30 min using electrodes measuring 25–35 cm^2^, resulting in current densities of 0.03–0.08 mA/cm^2^ (62–64). Treatment generally consists of 5–10 consecutive daily sessions, although repeated treatment cycles over several weeks have also been investigated (57). The cathode is usually positioned over the presumed epileptogenic cortex, whereas the anode is placed over the contralateral supraorbital region or an extracephalic reference site (64, 65). Advances in computational brain modeling have enabled more precise characterization and optimization of intracerebral electric fields, moving beyond conventional two-electrode montages toward multi-electrode configurations in which current amplitudes can be individually adjusted to improve focality and target engagement (66). In DRE, this modeling-based approach has recently been extended to personalized multichannel tDCS, with electrode positions and current distributions optimized according to patient-specific epileptogenic networks and biophysical head models (67). Despite these commonly adopted parameters, electrode montages, treatment schedules, and stimulation dose remain heterogeneous, underscoring the need for standardized protocols.

##### 3.2.2.2 Transcranial direct current stimulation: clinical evidence

c-tDCS currently has one of the strongest evidence bases for DRE. A recent systematic review and meta-analysis including 267 patients with focal DRE demonstrated significant and consistent electrophysiological as well as clinical benefits following c-tDCS (1–2 mA, 20–30 min). Active stimulation significantly reduced IEDs at 2 weeks (mean difference: 6.14; *n* = 42), 4 weeks (5.44; *n* = 110), and 8 weeks (3.57; *n* = 56). These neurophysiological improvements were accompanied by significant reductions in seizure frequency. The meta-analysis showed absolute reductions of 4.06 seizures/month at 4 weeks and 2.66 seizures/month at 8 weeks compared with sham, while individual trials reported relative seizure reductions ranging from approximately 42 to 90%, depending on the stimulation protocol and patient population. Notably, treatment effects were highly consistent across studies, with minimal statistical heterogeneity, supporting the robustness of these findings (62).

More recently, a prospective open-label study evaluated personalized multichannel SEEG-guided tDCS in 16 patients with focal DRE. Overall seizure frequency decreased by 20%, with six patients meeting responder criteria. Among responders, mean seizure reduction reached 68%, with greater benefit observed in younger individuals and patients with extratemporal epilepsy. Although quantitative IED outcomes were not reported, the study demonstrated the feasibility, safety, and potential clinical value of individualized, network-guided stimulation strategies (67).

Across randomized trials, c-tDCS has consistently demonstrated an excellent safety profile in both pediatric and adult populations. Reported adverse events are mild, transient, and generally limited to scalp discomfort, itching, erythema, or headache, resolving spontaneously without treatment interruption (54).

#### 3.2.3 Transcranial alternating current stimulation: mechanism of action, stimulation parameters and clinical evidence

This technique acts by entraining endogenous cortical oscillations through externally applied alternating electrical currents (68). This mechanism is particularly relevant to epilepsy because pathological high-frequency oscillations (HFOs) represent established biomarkers of the seizure onset zone. Experimental evidence suggests that tACS can influence the frequency-specific organization and synchronization of these oscillatory networks (69, 70).

Stimulation parameters remain heterogeneous across tACS protocols, including stimulation frequency, intensity, electrode montage, session duration, and phase relationship with endogenous neural activity (53, 71). Because the therapeutic rationale of tACS in epilepsy is based on modulating pathological network oscillations, rather than solely altering cortical excitability, the selection of stimulation frequency and phase may be particularly important for target engagement. However, optimal parameters for seizure control remain to be established.

Despite this strong physiological rationale, clinical evidence remains limited. A randomized pilot trial found no significant reduction in seizure frequency compared with sham stimulation in patients with focal DRE (71). Rather than questioning the therapeutic concept itself, these findings likely reflect the marked heterogeneity of epileptic networks and the limitations of applying uniform stimulation protocols across biologically diverse patients. Consequently, recent research has shifted toward individualized stimulation strategies based on patient-specific electrophysiological signatures and network dynamics (72). Although tACS remains a promising approach for targeting pathological brain oscillations, its clinical efficacy in DRE will require research validation.

#### 3.2.4 Transcranial temporal interference stimulation: mechanism of action, stimulation parameters and clinical evidence

tTIS is the most recently developed NIN technique included in this review, first described in 2017 as a strategy to reach deep brain structures without directly activating the overlying cortex (73). Unlike conventional tES modalities, it delivers two or more high-frequency alternating currents, typically in the kHz range, through separate pairs of scalp electrodes. Because neuronal membranes filter frequencies above approximately 1 kHz, the carriers do not themselves evoke neural responses; their spatial interference instead generates a low-frequency amplitude-modulated envelope, tunable to DBS frequencies used in epilepsy such as 130 Hz, that engages deep neurons while relatively sparing the superficial cortex (74–76). Computational models can incorporate individual anatomical features to estimate the intracranial electric-field envelope, optimize focality and intensity, and steer the stimulation locus by adjusting the relative contribution of different electrode pairs without physically repositioning the electrodes (73, 77). This modeling-based approach is particularly relevant to multi-contact tTIS, in which independent control of multiple current sources may allow more precise field steering and allocation while reducing unintended stimulation of non-target regions. Importantly, population-level optimization has also been shown to approximate individualized montage optimization, potentially reducing reliance on patient-specific MRI while preserving targeting performance (77). These developments support a transition from fixed electrode configurations toward more individualized and computationally guided tTIS paradigms, although their clinical relevance in epilepsy remains to be established (78).

Clinical evidence of tTIS specifically in patients with DRE is currently limited to a single study. A registered, multicenter, sham-controlled study conducted across three centers provided the first clinical evidence of tTIS in patients with DRE. Thirteen adults (5 women and 8 men, aged 25 to 49 years) with focal DRE were studied while hospitalized with SEEG electrodes implanted for presurgical evaluation. Stimulation was delivered non-invasively through four scalp electrodes generating a 130 Hz amplitude-modulated envelope, using either lower (1 and 1.13 kHz) or higher (9 and 9.13 kHz) carrier frequencies at up to 2 mA per channel, in single 20-min sessions performed 6 to 10 days after electrode implantation. Active stimulation significantly reduced IEDs within the hippocampal focus, with a mean decrease of approximately 59% from baseline and individual reductions reaching 86%, accompanied by a parallel reduction in pathological high-frequency oscillations, particularly fast ripples. This suppression persisted for at least 20 min after stimulation ended, although discharge rates had returned to baseline values by 24 h. Two patients receiving the lower carrier frequency reported transient scalp tingling, and no adverse events were recorded. The effect of tTIS on seizures remains to be established through longitudinal studies in larger populations. The available clinical evidence therefore represents an early, biomarker-based proof of concept obtained during a single stimulation session in a presurgical inpatient setting, rather than a demonstration of antiseizure efficacy (79)3.3 Transcutaneous Vagus Nerve Stimulation.

### 3.3 Transcutaneous Vagus nerve stimulation

#### 3.3.1 Introduction and historical background

tVNS encompasses non-invasive approaches that electrically stimulate vagal afferent fibers through surface electrodes, primarily using either the auricular or cervical route. It was developed as a non-invasive alternative to implanted VNS, aiming to engage vagal neuromodulatory pathways while avoiding surgical implantation and device-related morbidity (80–82, 146). Following FDA approval of invasive VNS for DRE in 1997 and treatment-resistant depression in 2005, tVNS emerged as a more accessible strategy for vagal neuromodulation without the need for surgical implantation (80–82).

Two principal approaches are currently used: transcutaneous auricular vagus nerve stimulation (taVNS), which targets the auricular branch of the vagus nerve (ABVN) in the external ear, and transcutaneous cervical vagus nerve stimulation (tcVNS), which applies surface stimulation over the cervical vagus nerve. Although both approaches ultimately aim to engage central vagal afferent pathways, their anatomical access, peripheral fiber recruitment, and stimulation parameters differ and should therefore be considered separately (83) (Figure 3). Continuous technological advances have transformed early laboratory devices into compact, portable stimulators suitable for home-based therapy, expanding clinical applications beyond epilepsy (84, 85).

**Figure 3:**
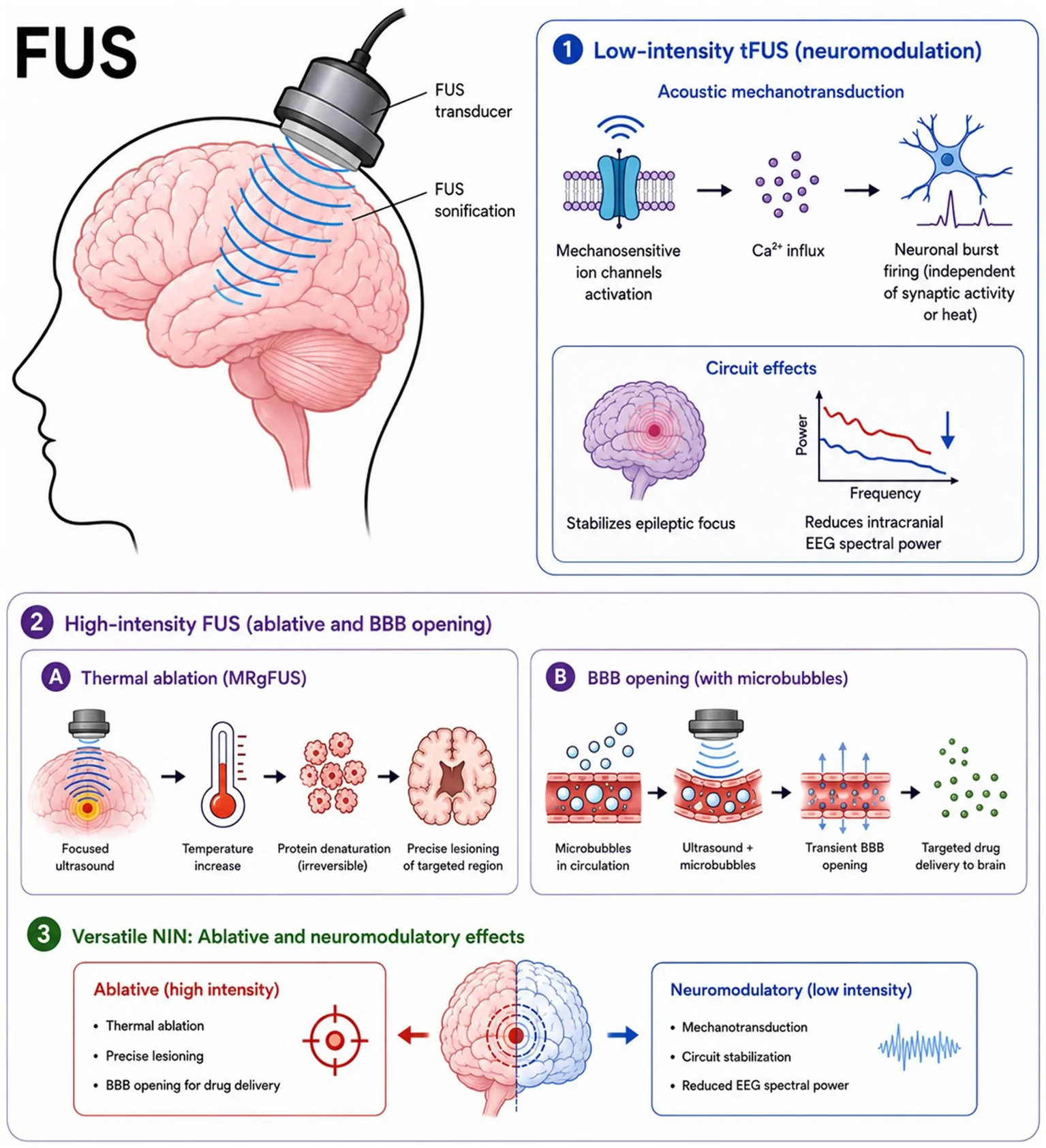
Proposed mechanisms of tVNS in epilepsy. **(A)** taVNS: a stimulation electrode or ear clip is positioned on the cymba conchae or tragus, regions with high vagal nerve fiber density. Stimulation of the cymba conchae (1) activates the auricular branch of the vagus nerve (ABVN) (2), transmitting afferent signals to the nucleus tractus solitarius (NTS), the primary sensory relay of the brainstem. From the NTS, neural pathways project to the locus coeruleus (LC) and dorsal raphe nucleus (DRN), promoting the release of norepinephrine and serotonin (3), which modulate cortical excitability and increase seizure threshold (4). **(B)** tcVNS: stimulates the cervical branch of the vagus nerve in the neck using surface electrodes, providing a more direct access to the nerve.

#### 3.3.2 Principles and mechanism of action

##### 3.3.2.1 taVNS

taVNS stimulates the auricular branch of the vagus nerve, particularly through regions of the external ear such as the cymba conchae, with afferent projections to the nucleus tractus solitarius (NTS). Activation of these pathways is followed by engagement of brainstem neuromodulatory systems, including the locus coeruleus (LC) and dorsal raphe nucleus (DRN), promoting the release of norepinephrine and serotonin and potentially increasing seizure threshold (86, 87). Beyond neurotransmitter modulation, taVNS has been associated with changes in autonomic balance, including increased parasympathetic activity and reduced sympathetic tone, as reflected by changes in heart rate variability (86, 88). It also activates the cholinergic anti-inflammatory pathway, suppressing pro-inflammatory cytokines such as TNF-α through nicotinic acetylcholine receptor signaling (89). Experimental evidence further suggests that tVNS promotes brain-derived neurotrophic factor release, facilitating synaptic plasticity and functional reorganization of epileptic networks, thereby providing a biological basis for sustained seizure control (88).

##### 3.3.2.2 tcVNS

tcVNS stimulates the cervical vagus nerve directly through surface electrodes placed over the neck. Compared with taVNS, this approach provides more direct anatomical access to the cervical vagus and may recruit a different distribution of vagal afferent fibers. Cervical stimulation engages ascending vagal pathways to the NTS and subsequently distributed brainstem neuromodulatory networks, including noradrenergic and serotonergic systems (90). Although both approaches converge on central vagal pathways and may produce similar population-level brainstem activation, their peripheral anatomy and patterns of neural recruitment are not identical. Accordingly, findings obtained with taVNS should not automatically be extrapolated to tcVNS, particularly with respect to optimal stimulation parameters and individual physiological responses.

#### 3.3.3 Stimulation protocols and technical parameters

##### 3.3.3.1 taVNS

taVNS protocols typically place an electrode over the cymba conchae or another auricular region containing vagal afferent fibers, with a reference electrode positioned at a non-vagal region of the ear. Standard active protocols generally employ frequencies of 10–25 Hz, pulse widths of 200–250 μs, and current intensities individually adjusted below the pain threshold, typically ranging from 0.5 to 5.0 mA (6, 91). Treatment schedules remain heterogeneous but commonly consist of 20-30-min sessions administered two to three times daily, with some protocols extending stimulation to 4 h/day. Clinical improvement is generally observed after approximately 20 weeks of consistent treatment. Sham stimulation most often uses 1 Hz to preserve blinding while minimizing physiological effects (6, 91). Despite increasing clinical experience, optimal stimulation parameters have yet to be standardized.

##### 3.3.3.2 tcVNS

tcVNS uses surface electrodes positioned over the cervical vagus nerve, allowing direct stimulation of the nerve through the neck. Clinical protocols remain less standardized than those used for taVNS, and the optimal stimulation intensity, frequency, pulse duration, treatment duration, and laterality remain incompletely defined. In the most recent randomized clinical study in DRE, bilateral cervical stimulation was administered for 2-min sessions three times daily over 12 weeks using a pre-programmed device (90).

#### 3.3.4 Clinical evidence in DRE

##### 3.3.4.1 taVNS

In adults, a recent meta-analysis reported responder rates ranging from 29–42%, with mean seizure reductions of approximately 30–65%, depending on stimulation parameters and follow-up duration (92, 93). Seizure freedom has been reported in up to 24% of patients, although superiority over sham stimulation has not been consistently demonstrated (94, 95). The overall responder rate reported by the most recent systematic review was 29% (11). Main outcomes in adult studies are summarized in Table 3.

**Table 3:** tVNS main outcomes among different studies.

Study (Author, Year)	Design of the study	Patient cohort (*n*)	Mean age (years)	Men/Women (*n*)	Epilepsy type (Focal/Generalized)	Stimulation protocol	Key outcomes
Aihua et al. (142)	RCT	60 patients (30 treatment, 30 control)	34.5 (16–60)	22/25 (of the 47 who completed)	32 simple partial, 6 complex partial, 9 generalized	Bilateral t-VNS; 20 Hz; 0.2 s pulse width; Intensity adjusted (+2 mA steps); 20 min, 3 times per day, for 12 months	Mean monthly seizures decreased from 8.0 to 4.0 in the treatment group.
Bauer et al. (91)	Randomized, double-blind, controlled trial	76 patients (37 treatment, 39 control)	38.8 ± 12.5	31/45	54 focal, 22 generalized.	Left taVNS; 25 Hz (active) vs. 1 Hz (control); 250 microseconds; 4 h daily for 20 weeks.	25 Hz group showed 23.4% mean reduction overall; completers (*n* = 26) had significant 34.2% reduction (*p* = 0.034).
Barbella et al. (98)	Open-label Pilot Study	20	38,6 (16–57)	10/10	Focal	taVNS was applied 4 h/day for 6 months, followed by a 2-month washout. Responders then received 2 h/day for 6 months. Intensity was 0.6–0.8 mA.	30% of patients (6/20) were classified as responders. In this specific group, a mean seizure reduction of 60% was achieved by the end of the study.
Liu et al.(6)	Prospective Open-label Study	17	27 ± 9.4 (12–65)	10/7	9 focal, 5 generalized, 3 both	Bilateral taVNS for 20 min, three times daily over 6 months. Stimulation parameters (10 Hz, 200 μs, 4 mA	16 out of 17 patients (94.1%) showed seizure reduction at 6 months, mean seizure reduction was 64.4%.
Gonzalez-Fernandez et al. (99)	Pilot Feasibility Study	18	29.94 (18–65)	9/9	Focal	taVNS left: 60 min, 3×/day for 6–9 months; minimal settings (25 Hz, 200 μs, 1.6–2.6 mA).	The study reported an 83.33% device sensitivity and confirmed clinical feasibility for DRE monitoring, while achieving a seizure reduction of over 50% in those classified as responders.
Madda et al. (90)	RCT	120 (60 treatment, 60 control)	Not reported	Not reported	Not reported	nVNS bilaterally on the neck for 2-min sessions, three times daily, for 12 weeks with device pre-programmed electrical pulses. The control group followed the same schedule using a sham.	45% median seizure reduction in the intervention group.

taVNS: Transcutaneous auricular vagus nerve stimulation. nVNS: Non-invasive vagus nerve stimulation (cervical). DRE: Drug-resistant epilepsy. RCT: Randomized controlled trial. n: Number of participants. μs: Microseconds (pulse width). mA: Milliamperes (current intensity). Hz: Hertz (cycles per second).

A systematic review identified 16 studies involving children and adolescents receiving taVNS, including one dedicated pediatric pilot trial and several mixed-age cohorts. Although stimulation protocols varied substantially, taVNS was consistently well tolerated, with favorable safety profiles. However, efficacy remains insufficiently established because of heterogeneous stimulation parameters, small pediatric cohorts, and the absence of age-specific protocol optimization (96).

Long-term adherence represents one of the principal advantages of taVNS. Because treatment is self-administered and readily incorporated into daily activities, it offers a practical home-based therapeutic option that may improve long-term compliance (96, 97). The clinical trials have not consistently demonstrated superiority of higher stimulation frequencies over conventional low-frequency protocols (91, 94, 98, 99).

##### 3.3.4.2 tcVNS

A recent RCT of 120 patients with DRE evaluated the efficacy of tcVNS. The stimulation protocol consisted of bilateral application on the neck for 2-min sessions, three times daily, over a 12-week period. At the end of the evaluation, the intervention group demonstrated a reduction in seizure frequency to 8.7 ± 3.9 seizures per month at 12 weeks compared with 15.8 ± 4.3 in baseline, corresponding to a 45% reduction in seizures compared with the sham group. Adverse effects were reported in 6 patients consisting in headache and transient neck discomfort. These findings suggest that tcVNS is a feasible, well-tolerated, and potentially effective modality for DRE management, although further studies are required to optimize long-term parameters (90).

### 3.4 Transcranial focused ultrasound stimulation

#### 3.4.1 Introduction and historical background

Focused ultrasound (FUS) has emerged as one of the most promising non-invasive neuromodulation technologies because it enables precise targeting of deep brain structures without surgical incision or implanted hardware (100). Although its earliest neurosurgical applications during the mid-twentieth century required craniotomy for movement disorders, advances in magnetic resonance guidance, acoustic modeling, and skull aberration correction have transformed FUS into a completely transcranial procedure with submillimeter targeting accuracy (101, 102). These technological developments have expanded its potential application to patients who are poor surgical candidates, including those at increased operative risk or receiving anticoagulant therapy (103).

#### 3.4.2 Principles and mechanism of action

The biological effects of FUS depend primarily on acoustic intensity. Low-intensity tFUS produces reversible neuromodulation through acoustic mechanotransduction, activating mechanosensitive ion channels and inducing calcium influx without generating clinically relevant tissue heating (104, 105). In epilepsy, these mechanical effects reduce cortical excitability, suppress intracranial EEG spectral power, and modulate epileptic networks, including deep subcortical structures that are difficult to access with other non-invasive techniques (100, 106).

By contrast, high-intensity MR-guided focused ultrasound (MRgFUS) generates irreversible thermal coagulation through protein denaturation, enabling stereotactic ablation of selected brain targets. When combined with circulating microbubbles, ultrasound can also produce transient and reversible blood–brain barrier opening, facilitating targeted drug delivery (107) (Figure 4).

**Figure 4:**
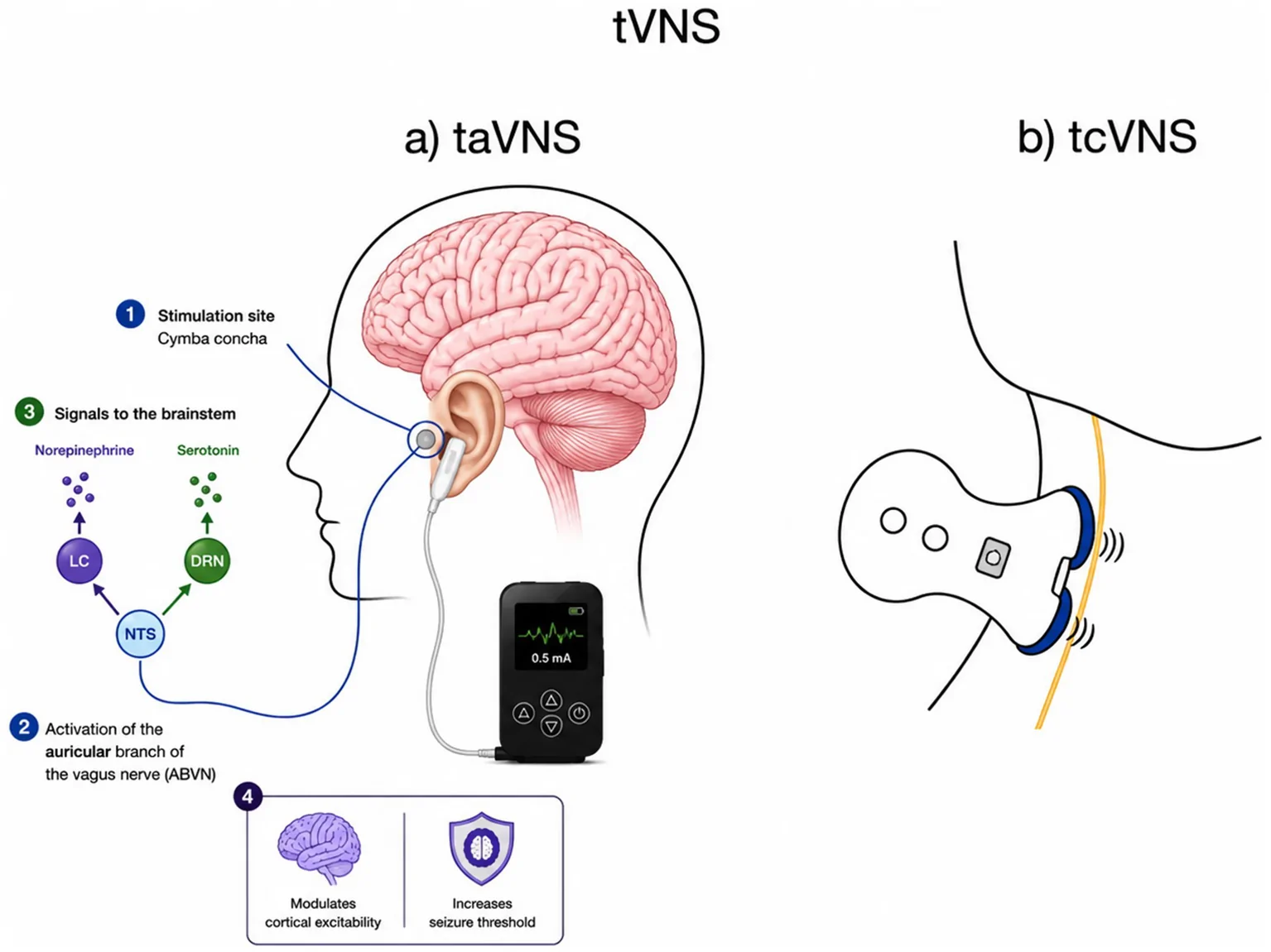
Principal mechanisms and therapeutic applications of FUS in epilepsy. The main component of the device is a hemispherical or phased-array transducer containing multiple ultrasound emitters. These transducers generate and focus acoustic energy on a specific intracranial target. Low-intensity transcranial FUS produces neuromodulatory effects through acoustic mechanotransduction, activating mechanosensitive ion channels, inducing calcium influx, and triggering neuronal burst firing independently of synaptic activity or thermal effects. tFUS may stabilize epileptic circuits and reduce intracranial EEG spectral power, thereby modulating seizure-related activity (1). In contrast, high-intensity FUS enables ablative effects through thermal energy deposition and irreversible protein denaturation, allowing precise lesioning of targeted brain regions. When combined with circulating microbubbles, FUS can also transiently open the blood-brain barrier to facilitate targeted drug delivery (2). Together, these mechanisms highlight the versatility of FUS as a non-invasive intervention with both neuromodulatory and ablative therapeutic potential in epilepsy (3).

#### 3.4.3 Stimulation protocols and technical parameters

Current tFUS protocols for DRE generally employ low-intensity sonication at 0.5 MHz with a 30% duty cycle, producing non-thermal neuromodulation. Experimental evidence indicates that prolonged stimulation sessions of approximately 10 min reduce SEEG spectral power within the seizure onset zone, reflecting transient suppression of neuronal excitability that persists beyond the stimulation period, although consistent reductions in seizure frequency have yet to be demonstrated (100).

In contrast, ablative FUS procedures use high-intensity MRgFUS to thermally lesion epileptogenic targets, including the anterior nucleus of the thalamus (ANT). To minimize cognitive complications, treatment planning maintains a 4–6 mm safety margin from the fornix (103, 108). Accurate energy delivery requires correction of skull-induced acoustic distortion using magnetic resonance-simulated computed tomography (MRsimCT), while real-time MRI thermometry and tractography further optimize targeting precision and procedural safety (102).

#### 3.4.4 Clinical evidence in DRE

Clinical evidence for FUS in epilepsy remains limited but continues to expand through early feasibility studies and first-in-human experiences. The strongest prospective initial evidence comes from a pilot study of six patients with DRE undergoing SEEG-guided low-intensity tFUS. Four patients demonstrated reduced IED frequency during the first 72 h, while two exhibited paradoxical electrophysiological activation. Clinically, seizure frequency improved in two patients, including one whose seizure burden decreased from 20 seizures/day at baseline to 10, 11, and 2 seizures/day during the first three post-treatment days. Although one participant experienced an apparent increase in seizure frequency, the additional events were exclusively subclinical, supporting the neuromodulatory effects of tFUS while highlighting interindividual variability (100, 109).

Beyond seizure control, preliminary studies indicate that tFUS can modulate pathological brain networks without disrupting surrounding tissue or altering blood–brain barrier integrity, whereas ablative MRgFUS offers the possibility of permanently interrupting epileptogenic circuits with stereotactic precision (100, 103, 106, 109). Nevertheless, current human evidence remains limited to early-phase investigations, precluding definitive conclusions regarding long-term efficacy, optimal treatment parameters, or patient selection.

Low-intensity tFUS has demonstrated an excellent short-term safety profile. Reported adverse events are generally mild and transient, consisting mainly of scalp heating, headache, or brief cognitive symptoms such as mild memory or naming difficulties that resolve spontaneously within several weeks (100).

### 3.5 Trigeminal nerve stimulation

#### 3.5.1 Introduction and historical background

External trigeminal nerve stimulation (eTNS) is delivered transcutaneously through adhesive electrodes positioned over the supraorbital and supratrochlear branches of the trigeminal nerve, providing a simple, portable, and non-invasive therapeutic (110, 111).

TNS originated from experimental observations demonstrating that trigeminal afferent stimulation could influence brainstem nuclei involved in seizure regulation, particularly through projections converging on the NTS and other neuromodulatory centers that overlap with vagal pathways (111). These findings encouraged the development of external stimulation devices capable of delivering repetitive electrical stimulation without requiring surgical intervention. During the last two decades, clinical investigations have progressively evaluated TNS as an adjunctive therapy for focal DRE (112). However, the current evidence remains less extensive than that available for other techniques.

#### 3.5.2 Principles and mechanisms of action

eTNS is administered non-invasively and bilaterally using adhesive cutaneous electrodes placed over the supraorbital branches of the trigeminal nerve on the forehead (113). It emerged as an alternative NIN modality aiming to target the same brainstem relay centers as VNS but through a different cranial nerve pathway. Mechanistically, the trigeminal nerve projects directly to the NTS in the brainstem, which represents the same target structure utilized in VNS (111, 113) (Figure 5).

**Figure 5:**
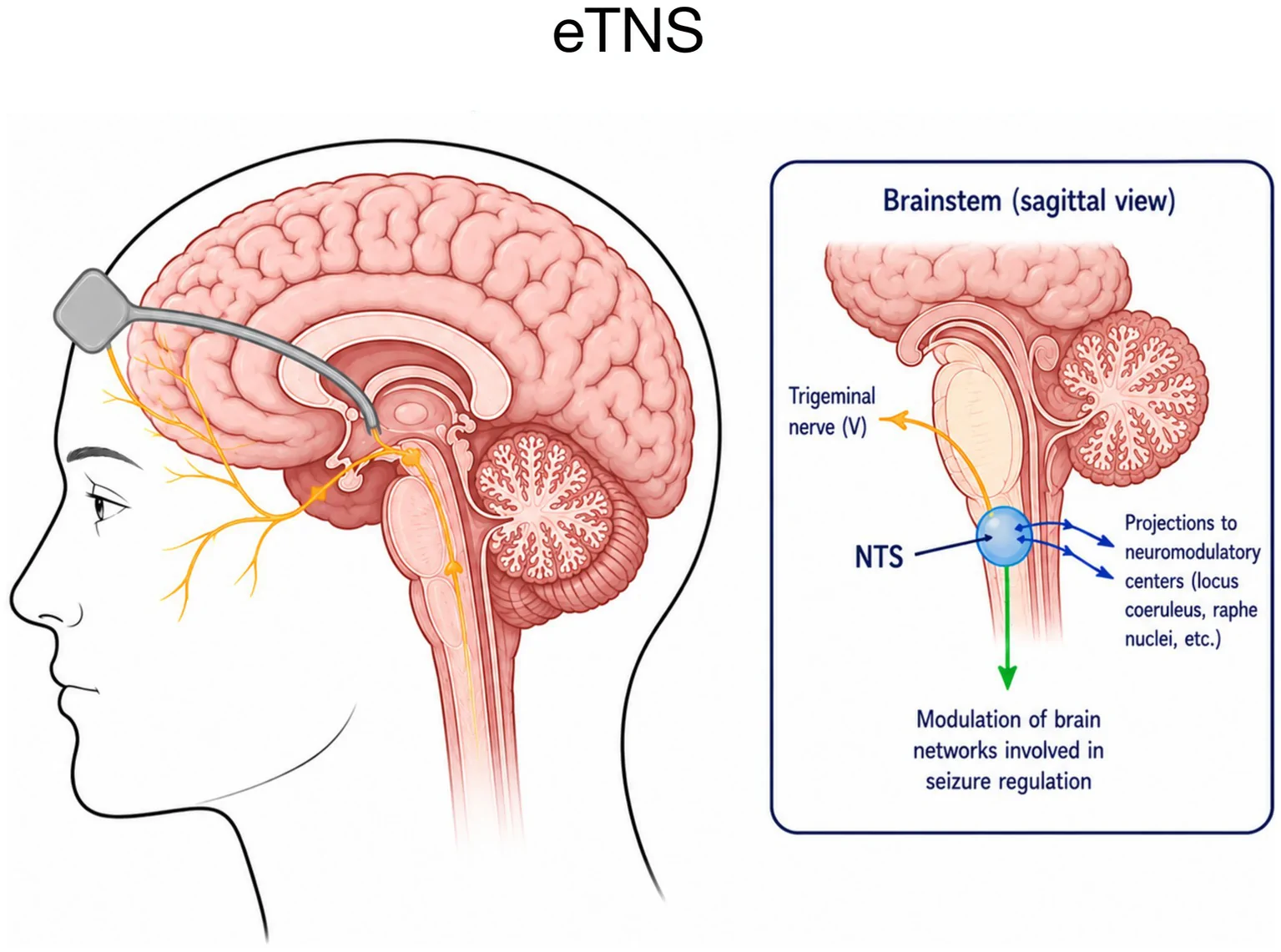
External trigeminal nerve stimulation (eTNS) is delivered transcutaneously through electrode placement over the supraorbital/supratrochlear branches of the trigeminal nerve.

#### 3.5.3 Stimulation protocols and parameters

Most clinical studies employ stimulation frequencies ranging from 100 to 120 Hz, pulse widths between 200 and 250 μs, and stimulation intensities individually titrated to produce a comfortable paresthesia without pain, commonly around 4–10 mA (113, 114). Treatment is generally administered during sleep for approximately 8–12 h per night, although daytime protocols have also been investigated (114). Despite encouraging clinical results, stimulation parameters remain heterogeneous, and optimal dosing schedules have not yet been established.

#### 3.5.4 Clinical evidence in DRE

A recent systematic review and meta-analysis which encompassed 9 studies and 411 patients confirmed that TNS significantly increased the short-term (3 months) responder rate at least two times compared with sham. In single-arm analyses, the proportion of responders was 42% patients at 3 months, 48% at 6 months, and 45% at 12 months, with a mean seizure reduction progressing from 29% at 3 months to 46% at 12 months (115).

TNS is well-tolerated with minimal adverse effects. The most common events are mild skin irritation and headache (116). No severe or life-threatening adverse events have been reported, nor has long-term TNS negatively affected neuropsychological function. Current limitations include the fragility of long-term comparative evidence, as statistically significant reductions versus control groups are most evident at 3 months, but confidence intervals widen at 6 and 12 months (115). Larger RCTs are needed to standardize protocols and confirm long-term efficacy.

Taken together, these modalities differ substantially in their biophysical mechanisms, target accessibility, spatial specificity, clinical evidence, and potential applicability across epilepsy phenotypes. Table 4 summarizes these features to provide an integrated framework for the clinical and translational positioning of NIN modalities in DRE.

**Table 4:** Biophysical and clinical positioning of non-invasive neuromodulation modalities in drug-resistant epilepsy.

Modality	Bioelectronic/Biophysical principle	Primary target and reach	Spatial specificity/Targeting	Main limitations	Most plausible current clinical niche	Current evidence level
rTMS	Electromagnetic induction generates intracortical electric currents that modulate cortical excitability and network activity. Low-frequency protocols are generally used to reduce cortical hyperexcitability.	Primarily superficial cortical targets, with downstream effects on connected epileptic networks.	Moderate–high when focal coils and neuronavigation are used; targeting depends on cortical accessibility and anatomical localization.	Limited penetration to deep structures; heterogeneous stimulation protocols, targeting strategies, and response across patients; limited long-term evidence.	Focal, well-localized cortical DRE, particularly when the epileptogenic cortex is accessible to stimulation. Evidence for generalized epilepsy remains insufficient.	Moderate, but heterogeneous; a 2026 systematic review/meta-analysis found modest seizure-frequency reductions at 4 and 8 weeks and rated the overall evidence as moderate by GRADE, with important study-level limitations (137); (46); (45)d.
tDCS	Weak direct electric fields alter neuronal membrane polarization, cortical excitability, synaptic plasticity, and network synchronization. Cathodal stimulation is most commonly investigated for DRE.	Predominantly cortical, with broader effects on connected networks rather than direct focal modulation of deep structures.	Low–moderate with conventional montages; potentially improved through high-definition and multi-electrode, model-guided stimulation.	Broad and anatomy-dependent current distribution; montage and dose remain heterogeneous; optimal parameters and durability are not yet standardized.	Focal DRE with an identifiable cortical target; currently one of the most clinically mature NIN approaches, particularly for adjunctive seizure reduction.	Moderate; multiple meta-analyses report significant seizure-frequency and IED reductions, although heterogeneity and small samples remain important limitations (118); (62); (122).
tACS	Alternating electric fields interact with endogenous oscillatory activity through frequency and phase-dependent modulation or entrainment of neural networks.	Primarily cortical and network-level oscillatory targets.	Moderate in principle, but target engagement is strongly dependent on frequency, phase, montage, and individual anatomy.	Limited clinical evidence; physiological responses may vary across brain states and patients; optimal frequency and phase are not established for epilepsy.	Potentially most relevant to focal or multifocal DRE with identifiable pathological oscillatory activity, but currently investigational.	Low/insufficient for clinical efficacy; small clinical studies have demonstrated feasibility but inconsistent physiological effects, with no robust seizure-frequency evidence (71); (56); (11).
tRNS	Broadband stochastic electrical stimulation may modulate neuronal excitability through stochastic resonance and nonlinear network effects.	Predominantly cortical, with potential network-level effects.	Low–moderate, depending on electrode montage; less target-specific than focal magnetic or ultrasound approaches.	Very limited epilepsy-specific clinical evidence; optimal frequency spectrum, dose, and target remain uncertain.	No epilepsy phenotype can currently be prioritized; potentially relevant to cortical network modulation once clinical target-engagement and efficacy data become available.	Very low/insufficient; current evidence is largely mechanistic or extrapolated from broader tES research, with insufficient clinical data to estimate seizure efficacy (129); (11).
taVNS	Peripheral vagal afferent stimulation activates brainstem nuclei, particularly the NTS, with downstream modulation of noradrenergic, serotonergic, and distributed cortical networks.	Peripheral-to-central, primarily engaging brainstem and distributed neuromodulatory networks rather than directly stimulating cortex.	Low for a discrete cortical target, but potentially broad network access through ascending vagal pathways.	Indirect and relatively diffuse central effects; substantial variability in auricular anatomy, stimulation site, dose, and treatment schedule.	Potentially attractive for DRE without a single readily accessible cortical target, including multifocal or distributed epileptic networks; also attractive when home-based treatment and avoidance of surgery are priorities.	Low–moderate; meta-analyses demonstrate promising responder rates and seizure reduction, but heterogeneity and limited long-term sham-controlled evidence remain substantial (92); (93); (11).
tcVNS	Electrical stimulation of cervical vagal afferents produces central neuromodulatory effects through ascending vagal pathways.	Peripheral-to-central, with access to brainstem and distributed networks.	Low for focal cortical targeting, but potentially more direct access to the cervical vagus than auricular stimulation.	Limited epilepsy-specific evidence and uncertainty regarding optimal stimulation parameters; long-term efficacy remains unestablished.	Potentially relevant to distributed-network DRE or patients for whom a peripheral, easily administered modality is preferable.	Low/emerging; a recent 120-patient randomized study reported a 45% seizure reduction over 12 weeks, but independent replication and longer follow-up are required (90).
tTIS	Two or more high-frequency electric fields interact to generate a low-frequency amplitude-modulated envelope at the intended target, potentially allowing deep neuromodulation without direct superficial activation.	Designed for deep structures, including mesial temporal/hippocampal networks, while relatively sparing superficial cortex.	Potentially high, particularly with individualized computational modeling and multi-contact current optimization.	Field strength at depth is limited; targeting depends strongly on anatomical modeling, electrode configuration, and current allocation; clinical evidence remains very limited.	Deep or mesial temporal DRE, particularly when a hippocampal or other deep target can be defined; currently one of the most mechanistically compelling emerging approaches for deep epilepsy targets.	Very low/early clinical evidence; a 13-patient human SEEG study demonstrated acute suppression of hippocampal epileptic biomarkers, but seizure efficacy and durability remain unestablished (131); (79); (130).
tFUS	Acoustic energy produces reversible neuromodulation through mechanotransduction and modulation of mechanosensitive ion channels, while higher-intensity FUS can produce focal thermal ablation.	Can reach deep and superficial structures with potentially millimeter-scale focality.	High theoretical spatial specificity, although skull attenuation, aberration, and acoustic modeling influence targeting accuracy.	Technical complexity, skull-related acoustic distortion, limited epilepsy-specific clinical evidence, and need for specialized equipment; LIFU and HIFU should not be considered interchangeable.	Deep, focal epileptogenic targets, particularly mesial temporal or other anatomically accessible structures; currently investigational for neuromodulation.	Very low /early clinical evidence; clinical LIFU experience in DRE remains limited to small pilot/feasibility studies, with insufficient data for pooled seizure efficacy (100); (109).
TNS	Peripheral trigeminal afferent stimulation activates brainstem and distributed neuromodulatory pathways, including NTS-related and locus coeruleus networks.	Peripheral-to-central, with indirect modulation of brainstem and distributed cortical networks.	Low for a discrete epileptogenic focus; effects are primarily network-mediated.	Indirect mechanism, heterogeneous stimulation protocols, and limited comparative and long-term randomized evidence.	Potentially useful for DRE in which distributed network modulation is desirable, particularly when focal cortical targeting is not feasible or when a peripheral modality is preferred.	Low–moderate, with fragile long-term evidence; a 2025 meta-analysis of nine studies (411 patients) found a significant response advantage at 3 months but not at 6 or 12 months (115); (11).

## 4 Discussion

### 4.1 Clinical and practical interpretation of NIN modalities

Beyond efficacy and safety, the clinical value of NIN is influenced by treatment burden, accessibility, and the feasibility of sustained use. These factors may substantially affect real-world effectiveness, particularly for therapies that require repeated sessions over weeks or months. rTMS generally requires specialized equipment, trained personnel, and repeated clinic visits, whereas tES and peripheral stimulation approaches can be delivered with portable devices and may be more suitable for home-based treatment (35–37, 47). taVNS has demonstrated favorable adherence in controlled settings, although real-world retention may be limited by device handling and integration into daily routines (91, 97). tcVNS and TNS may offer additional practical advantages through brief or predominantly passive treatment schedules, whereas LIFU currently requires substantially greater technical infrastructure and, in DRE, has largely been investigated in hospital-based, SEEG-guided settings (90, 100, 109, 113, 114) Thus, treatment burden and accessibility should be considered alongside seizure efficacy when evaluating the clinical maturity of each modality.

The duration of an NIN trial also remains an important unresolved clinical question. Therapeutic effects of rTMS, tDCS, and taVNS may evolve over weeks to months, making short observation periods potentially insufficient to determine treatment failure (7, 39, 62, 91) At the same time, adherence may limit the feasibility of prolonged trials, as demonstrated by the modest long-term retention reported in prospective taVNS studies (97). A related question is whether failure of one NIN modality should influence subsequent consideration of invasive neuromodulation. Current evidence does not support such an approach: response varies according to stimulation parameters, target localization, and network characteristics, and non-response to one modality does not establish resistance to a mechanistically distinct intervention (42). Similarly, there is no evidence-based sequence defining how many NIN approaches should be attempted before invasive treatment is considered. Until validated biomarkers and standardized treatment algorithms become available, NIN response should therefore not be used as a gatekeeping criterion that delays potentially effective invasive therapy (117).

### 4.2 Biophysical and clinical positioning of NIN modalities

NIN modalities differ substantially in how they interact with epileptic networks, and their therapeutic potential should therefore be interpreted in relation to target accessibility and the physical properties of each intervention. rTMS and tES primarily modulate cortical tissue and interconnected networks, with cathodal tDCS currently showing the most consistent clinical evidence among NIN approaches (62, 118). In contrast, tTIS and tFUS offer greater theoretical access to deep epileptogenic structures, although their clinical development remains at an early stage. tTIS is particularly attractive for deep or mesial temporal targets because computational modeling can steer the stimulation field toward predefined structures (73, 77), whereas tFUS provides potentially high spatial specificity but remains technically demanding and supported by limited clinical evidence (100, 109). Peripheral approaches such as taVNS, tcVNS, and TNS differ fundamentally because they modulate brain networks through afferent pathways rather than directly targeting a cortical epileptogenic zone, making them potentially relevant when epileptic activity is distributed or when a single accessible cortical target cannot be defined (93, 115).

The current evidence does not support a universal hierarchy among NIN modalities, but it does suggest a preliminary phenotype-based positioning. Focal, well-localized cortical DRE may be particularly suitable for low-frequency rTMS and cathodal tDCS, especially when stimulation can be guided by electrophysiological or imaging data. Deep or mesial temporal epileptogenic networks represent compelling targets for emerging approaches such as tTIS and LIFU, although their present evidence remains primarily based on biomarker, feasibility, or small pilot studies rather than established seizure efficacy (79, 100). For multifocal or distributed epileptic networks, peripheral neuromodulation such as tVNS or TNS may provide a conceptually attractive network-level strategy, although the available evidence remains heterogeneous (11, 115). Conversely, insufficient evidence currently exists to prioritize any specific NIN modality for generalized epilepsies. As summarized in Table 4, modality selection should therefore be guided by seizure phenotype, target accessibility, network organization, treatment burden, and the maturity of the supporting evidence.

The potential complementarity of these approaches is best understood at the mechanistic and network level rather than as an established clinical strategy. Different modalities may act on distinct levels of the epileptic network, from cortical excitability and pathological oscillations to deep structures and distributed neuromodulatory pathways. However, no adequately powered studies have established that combining NIN techniques produces superior seizure control. Future research should therefore evaluate complementary strategies within biomarker-defined and mechanistically characterized patient populations rather than treating NIN as a homogeneous therapeutic class (55, 119).

### 4.3 Clinical interpretation of IED reduction and its relationship to seizure control

Recent clinical and meta-analytic studies have reported heterogeneous effects of NIN on IEDs, ranging from significant reductions in IED burden to non-significant or variable responses across patients and modalities (Table 5). Overall, these findings indicate that several NIN approaches can modify IED activity, particularly rTMS and tDCS, although the magnitude and durability of these effects vary according to the modality, stimulation protocol, recording method, and timing of assessment. Importantly, a reduction in IED burden should not be interpreted as a direct surrogate of seizure control (62, 120).

**Table 5:** Recent quantitative evidence on interictal epileptiform discharges following non-invasive neuromodulation in drug-resistant epilepsy.

Modality	Most recent sufficiently informative evidence	Design/Population/Control	Stimulation parameters/Target	Baseline IED rate	IED assessment/Follow-up	IED reduction (%)	Main IED finding
rTMS	(42); (46)	Melus: crossover, randomized, single-blinded, sham-controlled RCT; 18 focal DRE patients. Walton: SR of 8 RCTs, 241 participants	0.5 Hz, 90% rMT, 3,000 pulses/session ×5 days; MRI-guided epileptogenic-focus targeting	NR	20-min EEG recordings; IEDs secondary outcome; 3-month washout	NR	Treatment effect −7.65 IEDs (95% CI − 16.44 to 1.14; *p* = 0.0839); not statistically significant. Walton: 3/4 IED-reporting trials showed significant reductions, but no pooled estimate was possible.
tDCS	(62)	SR/meta-analysis; 9 sham-controlled RCTs, 267 total patients	Predominantly cathodal tDCS; heterogeneous protocols	NR	EEG IEDs; 2, 4 and 8 wk	NR	2 wk: MD − 6.14 (95% CI − 8.98 to −3.83; *p* < 0.00001; n = 42); 4 wk: MD − 5.44(−7.79 to −3.08; *p* < 0.00001; n = 110); 8 wk: MD − 3.57(−6.14 to −0.99; *p* = 0.007; *n* = 56). All statistically significant; I^2^ = 0%.
tACS	(56)	Randomized sham-controlled SEEG study; 5 tACS patients	Personalized 10-Hz tACS, 20 min; maximum 2 mA; individualized target with highest spike rate	NR; 20-min SEEG baseline	Automated SEEG spike/IED quantification; baseline → sham → active → post-stimulation	NR	Only 3/5 tACS patients had ≥2 contacts with baseline spike rate >3 spikes/min. Responses were heterogeneous; 1/5 showed a significant spike-rate reduction, but the reduction was also present during sham, preventing attribution to tACS. No group-level percentage reduction was reported.
tRNS	(11)	Recent SR/meta-analysis of NIN in DRE	tRNS included among NIN modalities	NR	IED outcome not quantitatively synthesized	NR	No quantitative IED result identified for tRNS; therefore, no IED effect estimate can be reported.
tTIS	(79)	Human SEEG study; 13 patients with epilepsy	Hippocampal/mesial temporal targeting; 130-Hz AM using kHz carrier pairs	Individual baseline; group absolute rate NR	SEEG IEDs during stimulation, 20 min and 24 h	58.5 ± 27.8%	Relative IED reduction 58.5 ± 27.8%, with individual reductions up to 86.3%; significant during stimulation (*p* < 0.0001) and at 20 min post-stimulation (*p* < 0.0001); IED rate returned to baseline at 24 h.
tVNS/taVNS/tcVNS	(94); (143)	SR/meta-analysis; 9 clinical studies, 788 patients	Predominantly taVNS; heterogeneous protocols	NR	EEG outcomes; IED reduction not specifically quantified	NR	Meta-analysis found broader EEG changes with taVNS (OR 2.17, 95% CI 1.03–4.58), but this was not an IED-specific outcome. Therefore, quantitative IED reduction = NR.
tFUS/LIFU	(100)	Prospective Phase-I pilot; 6 focal DRE patients undergoing SEEG; uncontrolled	SOZ-targeted LIFU; 2.8 W/cm^2^, 30% duty cycle, 10 min	138–5,620 IEDs/day	Continuous SEEG during sonication and for 3 days afterward	NR	IED frequency decreased in 4/6 (66.7%) and increased in 2/6 (33.3%). No valid pooled percentage reduction reported. (NaviFUS)
TNS	(144)	Randomized real/sham study; 29 DRE patients	Bilateral infraorbital TNS; 1–20 mA, 120 Hz, 20 min	NR	Scalp EEG; 10 min pre-, 20 min during-, 10 min post-stimulation	NR	No significant effect on IED detection; the significant EEG effect was increased absolute alpha power (F₃₄,₆₈₀ = 1.748; *p* = 0.006), not an IED reduction.

Percentage reductions are reported only when explicitly provided by the original study; mean differences, odds ratios, or heterogeneous individual responses were not converted into percentage reductions. DRE, drug-resistant epilepsy; IED, interictal epileptiform discharge; rTMS, repetitive transcranial magnetic stimulation; tDCS, transcranial direct current stimulation; tACS, transcranial alternating current stimulation; tRNS, transcranial random noise stimulation; tTIS, transcranial temporal interference stimulation; tVNS, transcutaneous vagus nerve stimulation; taVNS, transcutaneous auricular vagus nerve stimulation; tcVNS, transcutaneous cervical vagus nerve stimulation; tFUS, transcranial focused ultrasound; LIFU, low-intensity focused ultrasound; TNS, trigeminal nerve stimulation; rMT, resting motor threshold; SEEG, stereo electroencephalography; NR, not reported.

IEDs and seizures represent related but distinct manifestations of epileptic network dysfunction. Suppression of interictal activity at a particular recording site may therefore occur without sufficiently altering the broader network responsible for seizure initiation and propagation. Differences in disease stage, epileptogenic network organization, stimulation target, individual response, and EEG recording duration may further contribute to discordant electrophysiological and clinical outcomes. Accordingly, IED reduction is better interpreted as evidence of network modulation or physiological target engagement than as a validated surrogate endpoint for clinical efficacy (120, 121).

Recent human studies illustrate this distinction particularly well. In tTIS, SEEG recordings demonstrated an acute reduction in hippocampal epileptic biomarkers, with IED reductions of 58.5 ± 27.8% and individual reductions of up to 86.3%; however, these effects diminished toward baseline within 24 h, and sustained seizure control was not demonstrated (79). Similarly, in a Phase I LIFU study of six patients with DRE undergoing SEEG, IED frequency decreased in four patients and increased in two, while seizure responses were inconsistent (100). These findings illustrate why acute electrophysiological modulation, although important for demonstrating target engagement, should not be equated with durable ant seizure efficacy.

Future trials should therefore prioritize longitudinal clinical outcomes, including seizure frequency, responder rate, seizure freedom, and quality of life, while incorporating standardized electrophysiological measures at pre-specified time points. Establishing whether an early reduction in IED burden predicts, accompanies, or remains independent of subsequent clinical benefit will be particularly important for emerging modalities such as tTIS and FUS (79, 100). Similarly, the absence of validated pretreatment biomarkers remains a major limitation. Seizure semiology, epileptogenic network localization, baseline EEG characteristics, connectivity measures, and structural imaging may inform target selection, but none has yet been sufficiently validated to reliably predict an individual patient’s response to NIN (120, 121). Post-hoc IED changes may nevertheless provide useful evidence that the intended network was engaged, but they should be considered mechanistic correlates rather than predictive biomarkers unless their relationship with subsequent clinical outcomes is demonstrated prospectively (120).

### 4.4 Comparative outcomes and clinical decision-making in invasive vs. non-invasive neuromodulation

Comparison between invasive and NIN treatments should first consider the maturity of the available evidence for each modality. Among the NIN techniques reviewed, cathodal tDCS currently demonstrates the most consistent clinical efficacy, with independent meta-analyses reporting seizure-frequency reductions of approximately 39–45% during the first 4–8 weeks of treatment (62, 118, 122), while taVNS shows a more modest but still meaningful pooled responder rate of 29–42% (92, 93). Although rTMS has shown encouraging results, reported responder rates vary widely (0–80%), suggesting that treatment efficacy depends heavily on patient selection, stimulation parameters, and target localization rather than on the technique itself (42). In contrast, evidence supporting tACS, tRNS, tTIS, and low-intensity FUS remains limited to pilot studies, early feasibility trials, or preclinical investigations and should therefore be considered exploratory rather than established clinical therapies (71, 79, 123).

Invasive neuromodulation nevertheless continues to provide greater long-term seizure control, with median seizure reductions of approximately 53–75% reported after 2–9 years of follow-up, compared with roughly 30–47% across currently established NIN modalities (9). Invasive therapies may also provide sustained improvements in quality of life and other long-term outcomes, but require surgical implantation and carry procedure-related risks. NIN therefore occupies a complementary rather than simply competitive position: its principal advantages include avoidance of implantation, favorable safety profiles, reversibility, and potential accessibility for patients who are poor surgical candidates or prefer less invasive treatment options (11, 124).

The difference in treatment dynamics is also clinically relevant. NIN generally requires repeated sessions and, for several modalities, produces its most evident effects during the weeks following treatment, whereas implanted devices provide continuous stimulation and may demonstrate progressive benefits over longer follow-up (46, 125). DBS and RNS may achieve higher rates of seizure freedom than VNS, although this must be considered alongside greater procedural complexity and surgical risk (117). Accordingly, treatment selection should not be based on efficacy estimates alone but should integrate seizure type, epileptogenic network localization, number and accessibility of seizure foci, comorbidities, treatment burden, patient preferences, access to specialized care, and tolerance for procedural risk (9, 117). Importantly, current evidence does not support using NIN as a required therapeutic step before invasive neuromodulation, nor does failure of one NIN modality establish failure of the broader neuromodulation strategy.

### 4.5 Future directions

Future progress in NIN for DRE will likely depend on three complementary principles: individualized targeting, adaptive stimulation, and biomarker-guided patient selection. Across modalities, adequately powered multicenter RCTs using standardized stimulation protocols, uniform outcome measures, and longer follow-up remain the highest priority to establish efficacy, durability, and safety (42, 98). Future clinical development should also prioritize phenotype-based stratification, with modality selection guided by target accessibility, epileptic network organization, and the maturity of supporting evidence. Focal, well-localized cortical DRE may be particularly suitable for low-frequency rTMS and cathodal tDCS, whereas deep or mesial temporal networks represent potential targets for tTIS and LIFU. Peripheral approaches such as tVNS or TNS may be more appropriate for multifocal or distributed networks, although evidence remains limited, and no NIN modality currently has sufficient evidence to support modality-specific prioritization for generalized epilepsy.

Optimization of stimulation parameters will remain essential across modalities. For rTMS, this includes frequency, intensity, cumulative dose, coil geometry, treatment schedule, and targeting accuracy; similarly, tES, tVNS, TNS, and FUS require systematic evaluation of stimulation intensity, frequency, montage or target, session duration, treatment repetition, and dosing. Prospective dose–response studies and standardized reporting frameworks will be necessary to define reproducible therapeutic windows for efficacy and safety. Integration of EEG biomarkers, structural imaging, and functional connectivity may further improve treatment precision by identifying patient-specific epileptic networks. This approach has already been illustrated by individualized SEEG-guided multichannel tDCS, which achieved a mean seizure reduction of 68% among responders, while also highlighting the potential contribution of individualized targeting to treatment response (40, 67). Maintenance protocols and multimodal therapeutic combinations also warrant further investigation (35, 39).

Within tES, patient-specific computational modeling, virtual brain twins, and AI-assisted electric-field optimization may further refine treatment planning, while closed-loop systems incorporating real-time EEG feedback could adapt stimulation to periods of increased seizure susceptibility and reduce unnecessary exposure (79, 119, 126). Emerging approaches such as tTIS may extend non-invasive stimulation to deep epileptic networks, particularly mesial temporal structures, whereas tRNS may provide an alternative strategy for disrupting pathological synchronization through stochastic resonance (127–130) Early studies of tTIS have demonstrated acute suppression of IEDs and pathological high-frequency oscillations, but their clinical significance and durability remain uncertain; ongoing multi-session studies will be important to determine whether these physiological effects translate into sustained clinical benefit (79, 131).

For tVNS, future development will likely emphasize adaptive stimulation, improved wearable devices, and AI-assisted parameter optimization to reduce interindividual variability and facilitate long-term home-based treatment (99, 132). FUS research should similarly focus on optimized and repetitive stimulation paradigms, improved acoustic targeting, MRI-based phase correction, and image-guided stereotactic navigation. Integration with transient blood–brain barrier opening may enable targeted drug delivery, while closed-loop FUS guided by electrophysiological biomarkers or intracranial recordings could facilitate individualized treatment and accelerate clinical translation (100, 102–104, 106, 133).

Finally, NIN may evolve beyond its role as a therapeutic intervention toward a tool for probing network modifiability before invasive treatment. Network biomarkers, including synchronizability, directed connectivity, and electrophysiological signatures, have shown potential for predicting outcomes after VNS, DBS, and RNS (134, 135). Early clinical or electrophysiological responses to NIN may provide exploratory markers of network modifiability and target engagement; however, their ability to predict long-term therapeutic response remains unvalidated. Whether such responses could identify patients most likely to benefit from subsequent invasive neuromodulation remains unknown and should be evaluated prospectively. We propose this as a testable translational hypothesis rather than an established clinical application. Prospective studies should therefore determine whether NIN responsiveness can identify patients most likely to benefit from continued non-invasive treatment or subsequent invasive neuromodulation, establishing whether NIN can serve both as therapy and as a functional biomarker for individualized treatment selection.

## 5 Limitations

This narrative review was based on a structured search of four databases, with study selection guided by relevance to the review objectives. Consistent with the aims of a narrative synthesis, no predefined protocol, formal risk-of-bias assessment, or evidence-certainty framework was applied. Consequently, while this review provides a comprehensive overview of current evidence and emerging developments in NIN for DRE, it may not have captured all relevant studies and does not support formal quantitative assessment of the strength or certainty of the available evidence.

The interpretation of the current literature is further limited by substantial heterogeneity across neuromodulation modalities, including differences in stimulation parameters, patient populations, study design, sham protocols, outcome definitions, and follow-up duration. In addition, much of the available evidence, particularly for emerging techniques such as tACS, tRNS, tTIS, low and high-intensity FUS, and tTNS, derives from small pilot studies, feasibility trials, or early clinical experience rather than adequately powered randomized controlled trials. Publication bias toward positive findings may also contribute to an overestimation of therapeutic effects, underscoring the need for larger, standardized multicenter studies.

Finally, comparisons across studies remain challenging because treatment outcomes are frequently based on patient-reported seizure diaries, which are inherently susceptible to recall bias and variable accuracy. This limitation is particularly relevant for focal seizures with impaired awareness and nocturnal seizures, which are more likely to go unrecognized than generalized tonic–clonic or focal aware seizures, potentially affecting the reliability of seizure frequency estimates and cross-study comparisons of treatment efficacy.

## 6 Conclusion

NIN techniques represent a promising and rapidly evolving therapeutic approach for DRE, offering the potential to modulate epileptic networks while avoiding the risks associated with invasive procedures. Although accumulating evidence supports their efficacy and favorable safety profile in selected patients, treatment responses remain heterogeneous, and robust long-term data are still needed. The future integration of these therapies into routine clinical practice will depend not only on demonstrating sustained efficacy, but also on improving patient selection, optimizing stimulation strategies, and delivering meaningful patient-centred outcomes, including quality of life, tolerability, and timely access to effective treatment. As evidence continues to mature, NIN is poised to become an increasingly personalized component of comprehensive epilepsy care.
