# Supplementary material for: Non-invasive neuromodulation for drug-resistant epilepsy: a contemporary structured review of mechanisms, clinical evidence and future directions
Source: Front Neurol. 2026 Sep 18;17:1950268. doi: 10.3389/fneur.2026.1950268 (PMC13631966; doi:10.3389/fneur.2026.1950268)
Supplement: Supplementary file 1 [file Data_Sheet_1.DOCX]

Supplementary Material

# 1 Supplementary Data

The complete search strategy is provided below:

*Pubmed*

("Epilepsy"[Mesh] OR epilepsy[tiab] OR epileptic[tiab]) AND ("Drug Resistant Epilepsy"[tiab] OR drug-resistant epilepsy[tiab] OR pharmacoresistant epilepsy[tiab] OR refractory epilepsy[tiab] OR medically refractory epilepsy[tiab] OR intractable epilepsy[tiab] OR drug resistant[tiab]) AND (("Transcranial Magnetic Stimulation"[Mesh] OR transcranial magnetic stimulation[tiab] OR repetitive transcranial magnetic stimulation[tiab] OR rTMS[tiab] OR low-frequency rTMS[tiab] OR high-frequency rTMS[tiab] OR theta burst stimulation[tiab] OR TBS[tiab] OR cTBS[tiab] OR iTBS[tiab] OR deep TMS[tiab] OR navigated TMS[tiab]) OR ("Transcranial Direct Current Stimulation"[Mesh] OR transcranial electrical stimulation[tiab] OR transcranial electric stimulation[tiab] OR tDCS[tiab] OR transcranial direct current stimulation[tiab] OR tACS[tiab] OR transcranial alternating current stimulation[tiab] OR tRNS[tiab] OR transcranial random noise stimulation[tiab]) OR ("Vagus Nerve Stimulation"[Mesh] OR transcutaneous vagus nerve stimulation[tiab] OR taVNS[tiab] OR tVNS[tiab] OR auricular vagus nerve stimulation[tiab] OR transcutaneous auricular vagus nerve stimulation[tiab]) OR (focused ultrasound stimulation[tiab] OR focused ultrasound neuromodulation[tiab] OR ultrasound neuromodulation[tiab] OR transcranial focused ultrasound[tiab] OR transcranial focused ultrasound stimulation[tiab] OR tFUS[tiab] OR low intensity focused ultrasound[tiab] OR low-intensity focused ultrasound[tiab] OR LIFU[tiab])) NOT (case reports[Publication Type] OR case report[ti]) NOT (animals[Mesh] NOT humans[Mesh])

*Web of Science*

(epilepsy OR epileptic) (Topic) and ("drug resistant epilepsy" OR "drug-resistant epilepsy" OR "pharmacoresistant epilepsy" OR pharmacoresistant OR "refractory epilepsy" OR "medically refractory epilepsy" OR "intractable epilepsy") (Topic) and ( ( neuromodulation OR "non-invasive neuromodulation" OR "non-invasive brain stimulation" OR "noninvasive brain stimulation" OR NIBS OR "transcranial magnetic stimulation" OR "repetitive transcranial magnetic stimulation" OR rTMS OR "low-frequency rTMS" OR "high-frequency rTMS" OR "theta burst stimulation" OR TBS OR cTBS OR itrs OR "deep TMS" OR "navigated TMS" OR "transcranial direct current stimulation" OR tDCS OR tACS OR tRNS OR "transcranial electrical stimulation" OR "transcranial electric stimulation" OR "transcranial alternating current stimulation" OR "transcranial random noise stimulation" OR "transcutaneous vagus nerve stimulation" OR tons OR taVNS OR "auricular vagus nerve stimulation" OR "transcutaneous auricular vagus nerve stimulation" OR "focused ultrasound" OR "focused ultrasound stimulation" OR "focused ultrasound neuromodulation" OR "ultrasound neuromodulation" OR "ultrasound brain stimulation" OR "transcranial focused ultrasound" OR "transcranial focused ultrasound stimulation" OR tFUS OR life OR "low intensity focused ultrasound" OR "low-intensity focused ultrasound" ) ) (Topic) not ("case report")

*Scopus*

( TITLE-ABS-KEY ( ( epilepsy OR epileptic ) ) AND TITLE-ABS-KEY ( ( "drug resistant epilepsy" OR "drug-resistant epilepsy" OR "pharmacoresistant epilepsy" OR "refractory epilepsy" OR "medically refractory epilepsy" OR "intractable epilepsy" ) ) AND TITLE-ABS-KEY ( ( "transcranial magnetic stimulation" OR "repetitive transcranial magnetic stimulation" OR rTMS OR "theta burst stimulation" OR TBS OR cTBS OR iTBS OR "deep TMS" OR "navigated TMS" OR "transcranial direct current stimulation" OR tDCS OR tACS OR tRNS OR "transcranial alternating current stimulation" OR "transcranial random noise stimulation" OR "transcutaneous vagus nerve stimulation" OR taVNS OR tVNS OR "auricular vagus nerve stimulation" OR "transcutaneous auricular vagus nerve stimulation" OR "focused ultrasound" OR "transcranial focused ultrasound" OR "focused ultrasound neuromodulation" OR tFUS OR LIFU ) ) AND NOT TITLE-ABS-KEY ( ( "case report" ) ) )

*Embase*

('epilepsy'/exp OR epilepsy:ti,ab OR epileptic:ti,ab) AND ('drug resistant epilepsy':ti,ab OR 'drug-resistant epilepsy':ti,ab OR pharmacoresistant:ti,ab OR 'pharmacoresistant epilepsy':ti,ab OR 'refractory epilepsy':ti,ab OR 'medically refractory epilepsy':ti,ab OR 'intractable epilepsy':ti,ab OR 'drug resistant':ti,ab) AND (neuromodulation:ti,ab OR 'non-invasive neuromodulation':ti,ab OR 'noninvasive neuromodulation':ti,ab OR 'non-invasive brain stimulation':ti,ab OR 'noninvasive brain stimulation':ti,ab OR NIBS:ti,ab OR ('transcranial magnetic stimulation'/exp OR 'transcranial magnetic stimulation':ti,ab OR 'repetitive transcranial magnetic stimulation':ti,ab OR rTMS:ti,ab OR 'low-frequency rTMS':ti,ab OR 'high-frequency rTMS':ti,ab OR 'theta burst stimulation':ti,ab OR TBS:ti,ab OR cTBS:ti,ab OR iTBS:ti,ab OR 'deep TMS':ti,ab OR 'navigated TMS':ti,ab) OR ('transcranial direct current stimulation'/exp OR 'transcranial direct current stimulation':ti,ab OR 'transcranial electrical stimulation':ti,ab OR 'transcranial electric stimulation':ti,ab OR tDCS:ti,ab OR tACS:ti,ab OR 'transcranial alternating current stimulation':ti,ab OR tRNS:ti,ab OR 'transcranial random noise stimulation':ti,ab) OR ('vagus nerve stimulation'/exp OR 'transcutaneous vagus nerve stimulation':ti,ab OR tVNS:ti,ab OR taVNS:ti,ab OR 'auricular vagus nerve stimulation':ti,ab OR 'transcutaneous auricular vagus nerve stimulation':ti,ab) OR ('focused ultrasound':ti,ab OR 'focused ultrasound stimulation':ti,ab OR 'focused ultrasound neuromodulation':ti,ab OR 'ultrasound neuromodulation':ti,ab OR 'ultrasound brain stimulation':ti,ab OR 'transcranial focused ultrasound':ti,ab OR 'transcranial focused ultrasound stimulation':ti,ab OR tFUS:ti,ab OR 'low intensity focused ultrasound':ti,ab OR 'low-intensity focused ultrasound':ti,ab OR LIFU:ti,ab)) NOT ('case report'/it OR 'case report':ti)
